# Pathological ultrastructural alterations of myelinated axons in normal appearing white matter in progressive multiple sclerosis

**DOI:** 10.1186/s40478-023-01598-7

**Published:** 2023-06-20

**Authors:** Wendy Oost, Allard J. Huitema, Kim Kats, Ben N. G. Giepmans, Susanne M. Kooistra, Bart J. L. Eggen, Wia Baron

**Affiliations:** 1 Department of Biomedical Sciences of Cells and Systems, Section Molecular Neurobiology, University of Groningen, University Medical Center Groningen, Groningen, the Netherlands; 2 MS Center Noord Nederland, University of Groningen, University Medical Center Groningen, Groningen, the Netherlands

**Keywords:** Electron microscopy, g-ratio, Human brain (bank), Mitochondria, Multiple sclerosis, Myelin, Nanotomy, Ultrastructure

## Abstract

Multiple sclerosis (MS) pathophysiology includes inflammation, demyelination and neurodegeneration, but the exact mechanisms of disease initiation and progression are unknown. A major feature of lesions is lack of myelin, which increases axonal energy demand and requires adaptation in number and size of mitochondria. Outside lesions, subtle and diffuse alterations are observed in normal appearing white matter (NAWM) and normal appearing grey matter (NAGM), including increased oxidative stress, reduced axon density and changes in myelin composition and morphology. On an ultrastructural level, only limited data is available on alterations in myelinated axons. We generated large scale 2D scanning transmission electron microscopy images (‘nanotomy’) of non-demyelinated brain tissue of control and progressive MS donors, accessible via an open-access online repository. We observed a reduced density of myelinated axons in NAWM, without a decrease in cross-sectional axon area. Small myelinated axons were less frequently and large myelinated axons were more frequently present in NAWM, while the g-ratio was similar. The correlation between axonal mitochondrial radius and g-ratio was lost in NAWM, but not in NAGM. Myelinated axons in control GM and NAGM had a similar g-ratio and radius distribution. We hypothesize that axonal loss in NAWM is likely compensated by swelling of the remaining myelinated axons and subsequent adjustment of myelin thickness to maintain their g-ratio. Failure of axonal mitochondria to adjust their size and fine-tuning of myelin thickness may render NAWM axons and their myelin more susceptible to injury.

**Graphical Abstract:**

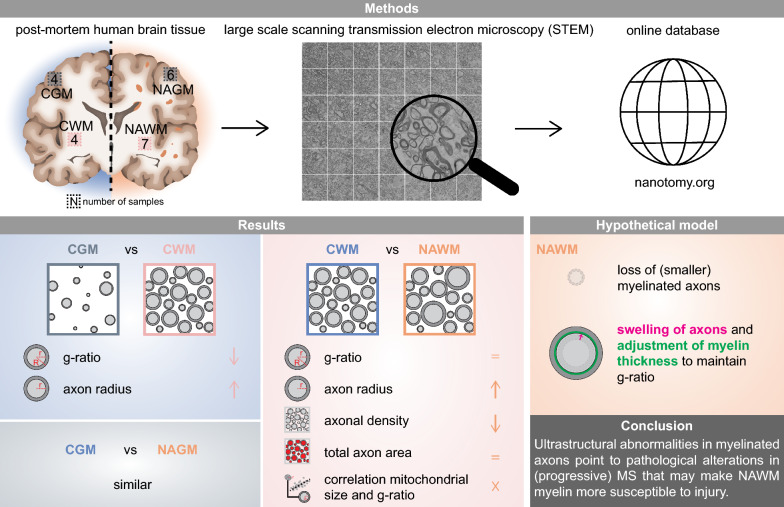

**Supplementary Information:**

The online version contains supplementary material available at 10.1186/s40478-023-01598-7.

## Introduction

Multiple sclerosis (MS) is a chronic neuroinflammatory and neurodegenerative disease of the central nervous system (CNS), in which demyelinated lesions occur in white and grey matter. Although non-demyelinated tissue appears macroscopically normal, it is referred to as normal appearing white (NAWM) or grey matter (NAGM), and neuroimaging, histopathological and biochemical studies showed that NAM differs from white (CWM) and grey matter (CGM) of control subjects. For example, NAWM and NAGM of relapsing remitting (RRMS) and progressive MS (SPMS/PPMS) contain magnetic resonance abnormalities that are more pronounced in NAWM [1–3]. In addition, the density of myelinated axons in NAGM [4] and NAWM [5] is reduced. Microglia are in a more activated state [6–10] and an increase in reactive oxygen species (ROS) causes oxidative damage in NAM [11]. Alterations in extracellular matrix components in NAWM contribute to a nonpermissive environment for axon regrowth and remyelination [12]. The widespread pathological changes in non-lesioned tissue are more prominent in late progressive MS, and in NAWM often correlate with lesion load and/or magnetisation transfer imaging (MTR) abnormalities [3, 13–15], indicating a potential secondary response to neurodegeneration.

Myelin structure and composition in NAWM are also abnormal. The nodes of Ranvier are disrupted, as evidenced by an increased overlap of the juxtaparanodes and paranodes [16–19]. In addition, MS myelin is biochemically different from myelin of control donors. Bulk analysis of myelin showed a shift in the lipid composition of NAWM to a higher phospholipid and lower sphingolipid content [20] as well as indication of changed lipid packing [21] and posttranslational alterations, namely citrullination and acylation, of myelin proteins like myelin basic protein [22, 23]. Next to these global changes in myelin composition, local blister-like swellings are formed by myelin detachment from axons [19, 24] and activated microglia clusters spatially accumulate in NAWM [25, 26], which may reflect early pathology of MS. Indeed, NAWM abnormalities in early RRMS display lesion-independent pathology [27], and MRI-based findings confirm that alterations in NAWM likely occur before lesion formation [15, 28, 29], which may relate to oligodendrocyte pathology or damage to the neuroaxonal unit.

An important structural property reflecting axonal function and integrity is the ratio of the inner and outer diameter of the myelin sheath (‘g-ratio’), which needs to be optimal for effective speed of impulse conduction [30, 31]. To comply with the altered energy demands upon demyelination and at a (sub)optimal g-ratio, axons adapt by adjusting the number and size of mitochondria and increase the speed of their transport [32, 33]. Remyelinated axons, hallmarked by thinner myelin sheaths, and therefore a higher g-ratio, have an increased mitochondrial content compared to myelinated axons [34]. Myelinated axons in NAWM have a lower mitochondrial content than myelinated axons in CWM [34]. In contrast, the number of mitochondria in myelinated axons is increased in optic nerve NAWM [18].

Identifying (sub)cellular and ultrastructural morphological changes of individual myelinated axons may help to understand pathological mechanisms in MS. Electron microscopy (EM) data on human MS brain tissue is very limited, and largely depends on studies from the 1960’s and 70’s that merely focused on lesions in small selected areas [35, 36]. The last decade higher throughput EM techniques provide more quantitative ultrastructural information of relatively large tissue areas (mm^2^ range) [37, 38]. Indeed, g-ratio analysis and the reported morphological changes in myelinated axons in NAM are just starting to be analysed at nanoscale resolution [18]. To obtain further insight into myelinated axonal pathology in NAM, we (1) determined ultrastructural features of myelinated axons and their mitochondria in CGM, NAGM, CWM and NAWM and (2) generated an open access database to vist and reuse the digitalized donor tissue in a Google Earth-like manner.

## Materials and methods

### Study design

Fresh post-mortem human brain tissue of progressive MS (NAWM, NAGM) and control donors (CWM, CGM) were processed for large scale STEM (nanotomy) and an EM database was created. The datasets were analysed for ultrastructural alterations of cross-sectional myelinated axons, axonal mitochondria, dendrites and dendritic mitochondria. For white matter, ultrastructural characteristics of 200 myelinated axons and their mitochondria were analysed per dataset, including density and total axon area of the myelinated axons. For grey matter, 100–200 myelinated axons and 200 dendrites and their mitochondria were analysed per dataset.

### Donors and sample collection

Fresh post-mortem white and grey matter brain tissue samples from donors with progressive MS and donors without neurological disease were obtained from the Netherlands Brain Bank (Table 1). Tissues were transported in cold HBSS with phenol red (Gibco, #14170-088) supplemented with 15 mM HEPES (Gibco, #15630-056) and 0.6% glucose (Sigma, #G8769). 4 control donors and 7 donors with progressive MS (mean disease duration 23 ± 4.2 years) were included in this study (Table 1). NAGM 7 was excluded because myelinated axons were absent. The male/female ratio for control donors was 3:1, and for donors with MS 4:3 for NAWM and 2:1 for NAGM. The median age of death of the control donors (77 ± 4.5 years) was significantly higher than the MS donors (61 ± 8.0 years for NAWM and 62 ± 8.7 years for NAGM). Post-mortem delay was comparable between control donors and donors with progressive MS. Informed consent was obtained by the Netherlands Brain Bank and the procedure approved by the Ethical Committee (Amsterdam UMC, the Netherlands).

**Table 1:** Donor information of white and grey matter human brain tissue samples

Donor^a^	Neur.^b^ diagnosis	Cause of death	Sex	M:F	Age	med ± SD^d^	PMD (h:min)^c^	med ± SD	Disease duration (years)	med ± SD
CM^e^ 1	–	Esophageal cancer	M	3:1	82	76 ± 4.0*	06:30	06:57 ± 1:51	–	
CM 2	–	Metastatic prostate cancer	M		77		07:25		–	
CM 3	–	Collapse, followed by asystole. probably cardiac death	M		73		10:15		–	
CM 4	–	Euthanasia	F		74		06:10		–	
NAM^f^ 1	PPMS	Respiratory insufficiency due to MS, assumed pneumonia	M	WM 4:3 GM 2:1	56	WM 61 ± 8.0* GM 62 ± 8.7*	06:15	WM 08:04 ± 1:24 GM 08:01 ± 1:32	20	WM 23 ± 4.2 GM 24 ± 4.4
NAM 2	SPMS	Euthanasia	F		66		09:30		25	
NAM 3	SPMS	Renal failure	M		53		06:25		24	
NAM 4	PMS	Sepsis	F		65		09:30		33	
NAM 5	SPMS	Euthanasia	M		58		08:59		23	
NAM 6	SPMS	Euthanasia	M		77		07:04		23	
NAWM^g^ 7	SPMS	Urosepsis, hydronephrosis	F		61		08:04		22	

*Median age of death was significantly higher in CM than in NAM (*p* < 0.05, Student’s *t* test)

^a^Color icons  are consistently used throughout the manuscript

^b^Neurological diagnosis

^c^Post-mortem delay from the Netherlands Brain Bank, excluding the transport time on ice (approx. 2 h) to the lab

^d^Median ± standard deviation

^e^Control matter

^f^Normal appearing (white and grey) matter

^g^normal appearing white matter

### Electron microscopy

Upon arrival, fresh white and grey matter brain tissue samples were cut into ~ 1–3  m^3^ pieces and fixated in cold 2% paraformaldehyde (PFA; Merck, #104005)-2% glutaraldehyde (GA; Polysciences, #01909) in 0.1M cacodylate buffer pH 7.4 (Sigma-Aldrich, #20840) and stored at 4 °C until further processing. Alternatively, 2% PFA-0.5% GA was used (dataset CM 1). After primary fixative (2–35 days, median of 6 days) samples were washed three times for 5 min with 0.1M cacodylate buffer and post-fixed in 1% osmium tetroxide (Electron Microscopy Sciences #19114) -1.5% potassium ferrocyanide (Merck, #P9387) in 0.1M cacodylate buffer for 2 h at 4 °C. After post-fixation, samples were washed four times for 5 min with MilliQ followed by dehydration and embedding in epoxy resin (glycid ether 100; SERVA, #21045, 2-Dodecenylsuccinic acid anhydride; SERVA, #20755, Methylnadic anhydride; SERVA; #29452, DMP-30; Polysciences, #00553). Semi-thin (~ 1 µm) sections were stained with toluidine blue to check if samples were homogenous in morphology. Samples were then trimmed to fit the grids, and ultrathin (~ 80 nm) sections were cut on an ultramicrotome with a diamond knife (Diatome, ultra 45°) and carefully positioned on formvar-coated copper L2 × 1 grids (Agar Scientific, #AGG2500C). Finally, sections were contrasted with 4% neodymium (III) acetate (Sigma-Aldrich, #325805) in MilliQ [39], washed five times with MilliQ and carefully dried and stored in a gridbox.

### EM acquisition and image processing

Image data were acquired on a Supra 55 scanning EM (SEM; Zeiss) using a scanning transmission EM (STEM) detector at 28 kV/25 kV with 2.5 nm pixel size with an external scan generator ATLAS 5 (Fibics) as previously described [40]. One dataset was made of 47 ± 21 tiles, one tile sized 16 k × 16 k pixels. The median dataset area is 70 k μm^2^. After image tile stitching, sample datasets were exported as html files and uploaded to the website https://www.nanotomy.org. Each dataset can be accessed at full resolution.

### Image analysis

#### Individual myelinated axons

From each dataset, 20 areas of 30 × 30 μm were exported from the html file at a resolution ~ 20 nm/pixel. The variation in shape and the frequency of touching axons in human brain tissue precluded the use of most publicly available (semi)automated tools, and preferred the use of the Gratio plugin tool from Fiji (http://gratio.efil.de/) [41, 42]. From each area, the tool randomly selected 10 cross-sectional myelinated axons per area from which the g-ratio was determined (n = 200 per donor for white matter, n = 100–200 per donor for grey matter). The analysis was performed by selecting the inner and the outer perimeter of the myelin sheath. The enclosed areas were used to automatically determine the g-ratio. The axon radius and myelin thickness were calculated from enclosed areas. Notably, the Gratio plugin tool does not take into account the inner tongue area, potentially leading to an overestimation of the axon radius. The compactness of the myelin sheath of the 200 measured axons was scored with a system ranging from 1 to 5, in which score 1 represents almost fully compact myelin and score 5 contains splits of the myelin layers along > 70% of the cross-sectional axon area. The presence of mitochondria in the myelinated axons was scored, and the cross-sectional mitochondrial size (area, radius, perimeter) and shape (circularity) were determined using Fiji.

#### Myelinated axon density and total myelinated axon area

From each white matter dataset, 25 areas of 20 × 20 μm were exported from the html file at a resolution around 20 nm/pixel. Areas without capillaries were selected, to prevent bias in the measurements. The number of myelinated axons and total axonal area, measured as the area enclosed by myelin, of each image were determined using Fiji.

#### Individual dendrites

From each grey matter dataset, the same areas used for analysis of individual myelinated axons were analysed for dendrites and their mitochondria. From each area, the dendritic area of 10 randomly selected dendrites was determined (n = 200 per donor) using the Gratio plugin tool from Fiji. The presence of mitochondria in the dendrites was scored, and the cross-sectional mitochondrial size (area, radius, perimeter) and shape (circularity) were determined using Fiji.

### Statistical analysis

Statistical analysis was performed using R (version 4.1.0, Integrated Development for R. RStudio, Inc., Boston, MA. URL http://www.rstudio.com/). A total of 2200 datapoints were available for statistical analysis of individual myelinated white matter axons (11 donors × 200 axons) and dendrites (11 donors × 200 axons). For the myelinated grey matter axons, 1501 datapoints were available (10 donors × 100–200 axons). For statistical analysis of individual mitochondria in myelinated axons, the number of available datapoints was; 143 of CWM (4 donors × 21–49 mitochondria), 92 of CGM (4 donors × 10–40 mitochondria), 101 of NAGM (6 donors × 6–31 mitochondria) and 269 of NAWM (7 donors × 27–69 mitochondria). For dendritic mitochondria 289 datapoints were available of CGM (4 donors × 54–101 mitochondria) and 333 of NAGM (6 donors × 44–72 mitochondria). For the axon density and total axon area, 275 datapoints were available (11 donors × 25 areas). Linear mixed-effect models were used in analysis to account for repeated measures. The nlme package in R was implemented with diagnosis as fixed variable and “donor ID” as a random factor. Including age and/or post-mortem delay (PMD) did not lead to a better model fit. For the comparisons in the percentage of axons/dendrites with mitochondria, the student’s *t* test was used to compare the means (calculated by taking the mean per donor). This test requires that samples are independent, of equal variance and normally distributed. Therefore, the Shapiro–Wilk test was used to confirm these assumptions prior to performing a Student’s *t* test. For the comparisons between control and MS donors, a Student’s *t* test was performed and for comparisons between control grey and white matter, a paired Student’s *t* test was performed. To test whether a linear correlation exists between the mitochondrial radius and the g-ratio, the Pearson correlation coefficient was used. Given the overrepresentation of male donors in our dataset, statistical difference for male donors only was analysed and indicated when significance deviate from the analysis of both sexes. For all statistical analyses *p* values < 0.05 were considered significant.

## Results

### Myelinated axons in CGM are smaller and have a higher g-ratio than myelinated axons in CWM

**Fig. 1:**
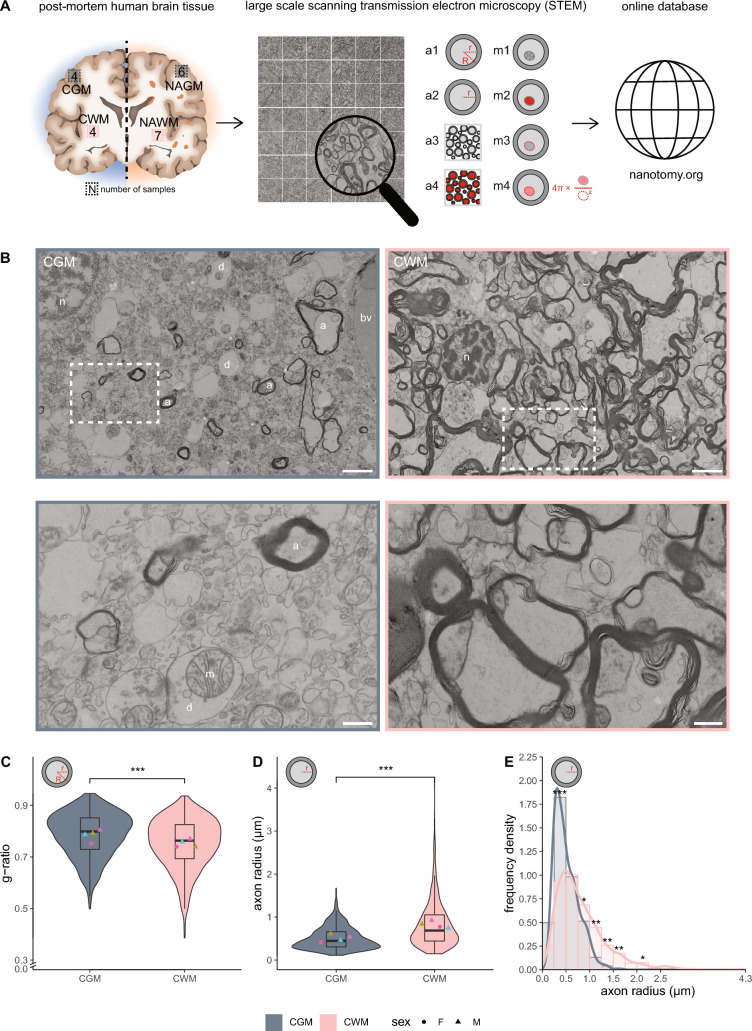
Myelinated axons in CWM have a lower g-ratio and a higher axon radius than in CGM. **A** Experimental set-up. Post-mortem human brain tissue samples were collected from control donors and donors with progressive MS and processed for large-scale scanning transmission electron microscopy (STEM). Datasets were analysed for ultrastructural features of myelinated axons (a1: g-ratio (r/R), a2: radius (r), a3: density; a4: total area) and their mitochondria (m1: number, m2: area, m3: perimeter, m4: circularity). Each dataset is accessible in full resolution via nanotomy.org. **B** Representative STEM images of post-mortem control grey matter (CGM) and control white matter (CWM) tissue of control donors (paired, from donor CM 1). Top: overview images. Bottom: detailed images from boxed regions in top images. a = myelinated axon, d = dendrite, n = nucleus, bv = blood vessel, m = mitochondria. Scale bar represents 2 µm (top images) or 0.5 µm (bottom images). **C**, **D** Violin plots depicting the distribution of g-ratio (**C**) and axon radius (**D**). Datapoints represent mean per donor (● = female, ▲ = male, color-coded, see Table 1) and boxplots show the median and inter quartile range (IQR). **E** Histogram with kernel density estimation of the axon radius (r). Number of control (CGM/CWM) donors is 4 (paired). For CGM 100–200 myelinated axons and for CWM 200 myelinated axons per donor were analysed. Statistics were performed using linear mixed model (**C**, **D**), or a paired sample Student’s *t* test (**E**) (*p < 0.05; **p < 0.01; ***p < 0.001)

Myelinated axons are more extensively studied in myelin-rich white matter than in myelin-poor grey matter tissue. Therefore, we first compared ultrastructural features of myelinated axons and their mitochondria in grey and white matter brain tissue of control donors (Fig. 1A). To this end, CGM and CWM from 4 non-demented control donors were analysed, in which the median dataset area was 70 k µm^2^ (Table 1). As expected, CGM contained less myelinated axons and more neuronal cell bodies and dendrites, while CWM contained mostly myelinated axons (Fig. 1B). As the g-ratio is an important structural trait that affects nerve impulse conduction, we next determined per donor the g-ratio of 200 myelinated axons from CWM and 150–200 myelinated axons from CGM. The g-ratio of myelinated axons in CWM was significantly lower than in CGM (CWM: 0.75 ± 0.015, CGM: 0.78 ± 0.022, *p* < 0.001) (Fig. 1C). In CGM, the measured radius of axons ranged from 0.11 to 1.7 μm, and in CWM from 0.15 to 4.2 μm. Furthermore, the radius of myelinated axons was higher in CWM than in CGM (CWM: 0.82 ± 0.090 µm, CGM: 0.51 ± 0.083 µm, *p* < 0.001) (Fig. 1D). Frequency density plots of axon radii indicate that CWM contained more large myelinated axons (0.75–1.75 µm) and less small myelinated axons (0.25–0.50 µm) compared to CGM (Fig. 1E). Hence, myelinated axons in CGM were smaller than myelinated axons in CWM. A higher g-ratio results in increased energy demands of axons to achieve optimal action potential propagation, which can be accomplished by alterations in mitochondria number and/or morphology [43]. Therefore, we next determined ultrastructural features of mitochondria in myelinated axons in CGM and CWM.

### Axonal mitochondrial radius in myelinated axons correlates with g-ratio in control matter

**Fig. 2:**
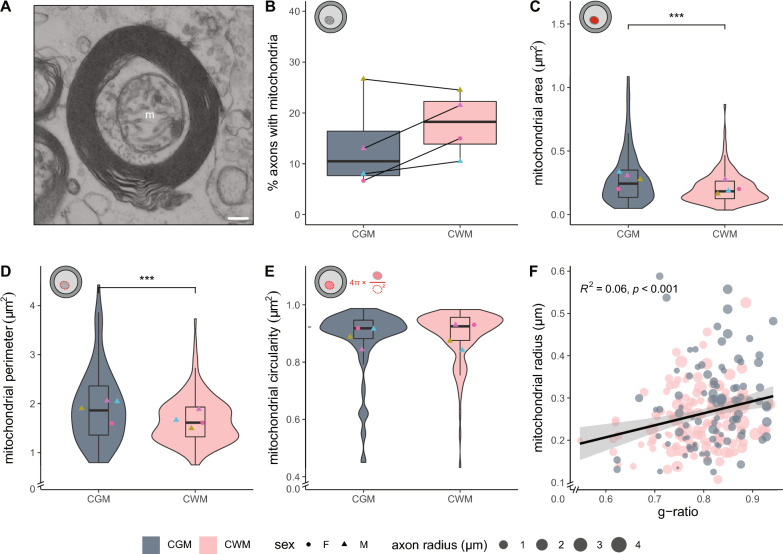
Mitochondrial size in myelinated axons correlates with g-ratio in control brain tissue. **A** Cross-sectional image of a myelinated axon with a mitochondrion (m) in post-mortem brain tissue of control donors. Scale bar represents 0.2 µm. **B** Mean percentage myelinated axons with mitochondria in post-mortem control grey matter (CGM) and control white matter (CWM) tissue of control donors. **C**–**E** Analysis of cross-sectional axonal mitochondrial area (**C**), perimeter (**D**), and circularity (**E**). Icons indicate measured mitochondrial characteristic (red), violin plots depict the data distribution of all measurements, datapoints represent mean per donor (● = female, ▲ = male, color-coded, see Table 1), and boxplots show the median and inter quartile range (IQR). **F** Scatter plot showing the correlation between mitochondria radius and g-ratio. The size of the datapoints indicates axon radius in µm. The number of control (CGM/CWM) donors is 4 (paired) and 10–49 mitochondria per donor were analysed (dependent on the number of mitochondria in the 100–200 myelinated axons that were measured in Fig. 1). Statistics were performed using a paired Student’s *t* test (**B**), linear mixed model (**C**–**E**), or a Pearson’s r test (**F**) (***p < 0.001)

To determine whether the higher g-ratio of myelinated axons in CGM may be compensated by alterations in their mitochondria, we determined the number, size and shape of mitochondria present in the myelinated axons analysed in Fig. 1 (Figs. 1A, 2A). The percentage of cross-sectional myelinated axons with mitochondria did not differ between CGM and CWM (Fig. 2B). The cross-sectional area of mitochondria that are present in myelinated CWM axons was smaller compared to the mitochondrial area in myelinated CGM axons, (CWM: 0.21 ± 0.049 µm^2^, CGM: 0.28 ± 0.058 µm^2^, *p* =  < 0.001) (Fig. 2C). The perimeter of mitochondria in myelinated CGM axons was significantly larger than in CWM (CGM: 1.9 ± 0.22 µm, CWM: 1.7 ± 0.16 µm, *p* < 0.001) (Fig. 2D). Cross-sectional mitochondria were similar in shape, as indicated by mitochondrial circularity (Fig. 2E). Overall, when the myelinated axons of CWM and CGM were combined, we observed a correlation between the mitochondrial radius and g-ratio (Fig. 2F), indicating homeostatic conditions [43]. To determine potential NAM ultrastructural abnormalities in myelinated axons and their mitochondria, we first compared CGM with NAGM.

### Myelinated axons in CGM and NAGM have a similar g-ratio and axon size distribution

**Fig. 3:**
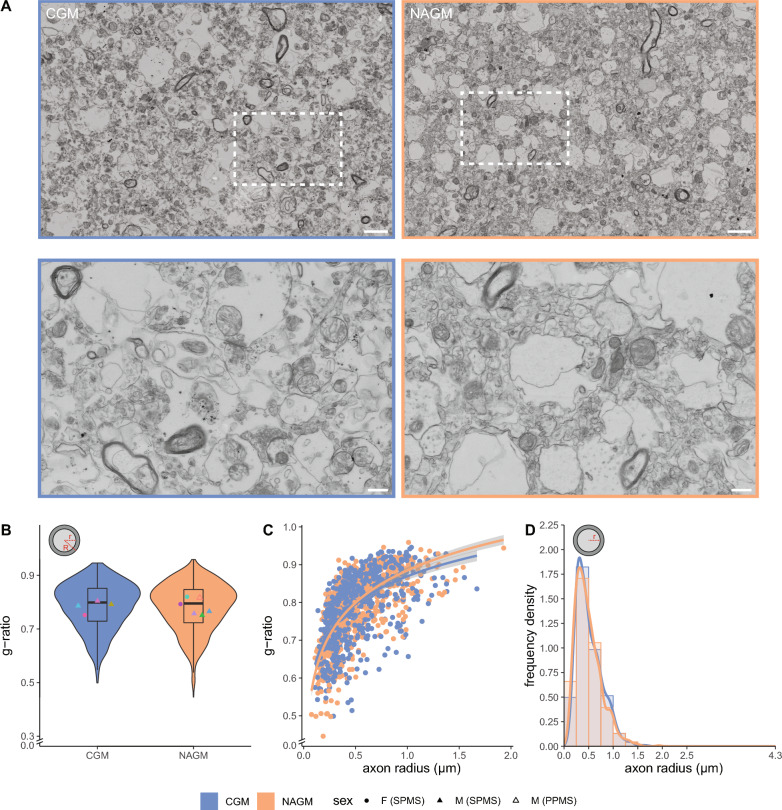
Myelinated axons in CGM and NAGM have a similar g-ratio and axon size distribution. **A** Representative scanning electron microscopic images (STEM) images of post-mortem control grey matter (CGM) of control donors (from donor CM 2) and normal appearing grey matter (NAGM) of donors with progressive MS (from donor NAM 6). Top: overview images. Bottom: detailed images from boxed regions in top images. Scale bars represent 2 µm (top images) or 0.5 µm (bottom images). **B** Violin plots depict the distribution of g-ratio (r/R). Datapoints represent mean per donor (● = female (SPMS), ▲ = male (SPMS), △ = male (PPMS), color-coded, see Table 1) and boxplots show the median and inter quartile range (IQR). **C** Scatter plot showing the g-ratio versus axon radius (log-linear fit). **D** Histogram with kernel density estimation of the axon radius (r). Number of control donors (CWM) is 4, number of donors with progressive MS (NAWM) is 6, and 100–200 myelinated axons per donor were analysed. Statistics were performed using a linear mixed model (**B**) or unpaired Student’s *t* test (**D**)

To establish whether ultrastructural pathological differences existed in myelinated axons in NAGM, we compared CGM from 4 non-demented control donors with NAGM of 6 donors with progressive MS (Table 1, Figs. 1A, 3A, 100–200 myelinated axons per donor). The onset of MS lesion formation is characterized by myelin decompaction and breakdown. Therefore, we first assessed myelin structure in CGM and NAGM using a scoring system based on the extent of (de)compaction (Additional file 1: Fig. S1A). The proportions of myelin compaction scores in NAGM were similar to the proportions in CGM (Additional file 1: Fig. S1B), indicating that there were no signs of substantial (early) demyelination in the analysed areas. Myelinated axons in CGM and NAGM had a similar g-ratio (Fig. 3B). The logarithmic regression curves in the g-ratio versus axon scatter plots did not completely overlay for larger axons (Fig. 3C). Larger myelinated axons in NAGM tended to have a higher g-ratio, which may point to remyelination, consistent with a previous study [44]. Axon radii analysis indicated no major difference between the distribution in CGM and NAGM (Fig. 3D). Hence, myelinated axons in CGM and NAGM have a similar g-ratio and axon radius distribution.

### Axonal mitochondrial radius correlates with g-ratio in NAGM

**Fig. 4:**
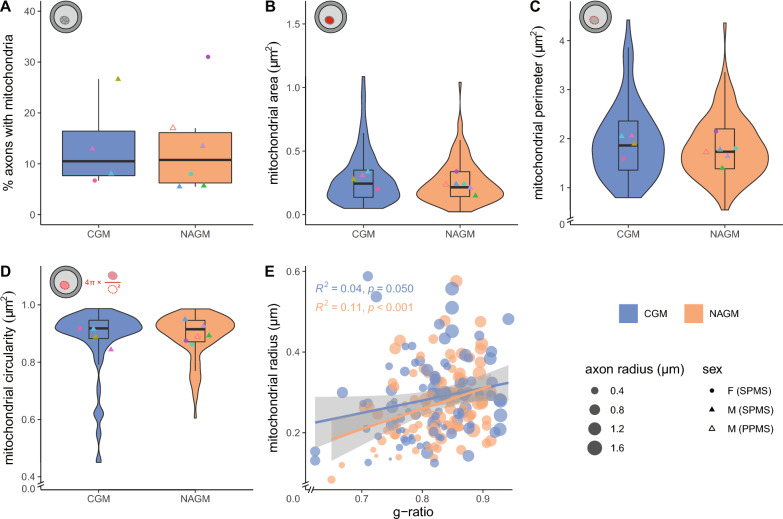
Axonal mitochondrial size correlates with g-ratio in NAGM. **A** Mean percentage myelinated axons with mitochondria in post-mortem control grey matter (CGM) of control donors and normal appearing grey matter (NAGM) tissue of donors with progressive MS. **B**–**D** Analysis of cross-sectional axonal mitochondrial area (**B**), perimeter (**C**), and circularity (**D**). Icons indicate measured mitochondrial characteristics (red), violin plots depict the data distribution of all measurements, datapoints represent mean per donor (● = female (SPMS), ▲ = male (SPMS), △ = male (PPMS), color-coded, see Table 1), and boxplots show the median and inter quartile range (IQR). **E** Scatter plot showing the correlation between mitochondria radius and g-ratio. The size of the datapoints indicates the axon radius in µm. Number of control donors (CGM) is 4, number of donors with progressive MS (NAGM) is 6, and 6–40 mitochondria per donor (dependent on the number of mitochondria in the 100–200 axons that were measured in Fig. 3) were analysed. Statistics were performed using an unpaired Student’s *t* test (**A**), linear mixed model (**B**–**D**), or a Pearson’s r test (**E**)

To determine whether potential alterations in energy supply by mitochondria exist, we determined whether mitochondria in myelinated axons in NAGM were changed. From the same myelinated axons that were measured in Fig. 3, we counted the number of myelinated axons with at least one mitochondrion and determined the cross-sectional mitochondrial area, perimeter and circularity (Fig. 4). The percentage of cross-sectional myelinated axons with mitochondria ranged from 5 to 31% and did not differ between CGM and NAGM (Fig. 4A). Axonal mitochondria in NAGM have a similar cross-sectional area and perimeter, (Fig. 4B, C), unless if for perimeter only male donors were compared (*p* = 0.039). The mitochondrial circularity was similar between CGM and NAGM (Fig. 4D). Furthermore, the mitochondrial radius and g-ratio correlated in NAGM (Fig. 4E), indicating homeostatic conditions. This correlation was not statistically significant within the *p* < 0.05 limit in CGM.

In addition to the analysis of myelinated axons and axonal mitochondria, we analysed the ultrastructural characteristics of 200 dendrites and their mitochondria in the same CGM and NAGM areas (Additional file 2: Fig. S2A). The total cross-sectional dendritic area was similar in CGM and NAGM (Additional file 2: Fig. S2B). Furthermore, the percentage of cross-sectional dendrites that contain mitochondria was similar in NAGM and CGM (Additional file 2: Fig. S2C, NAGM: 28 ± 5.4%, CGM: 36 ± 11%). Notably, the percentage of cross-sectional dendrites with mitochondria was higher than in myelinated axons (Additional file 2: Fig. S2C versus 4A, GM dendrites: 31 ± 8.7%, GM axons: 13 ± 9.0%, *p* < 0.001). The cross-sectional dendritic mitochondrial area, perimeter and circularity were similar in CGM and NAGM (Additional file 2: Fig. S2D–F). Thus, we observed no significant differences in cross-sectional axonal and dendritic mitochondria number, size and shape between CGM and NAGM.

### NAWM contains fewer myelinated axons that cover a similar cross-sectional axonal area as in CWM, while g-ratio is maintained

**Fig. 5:**
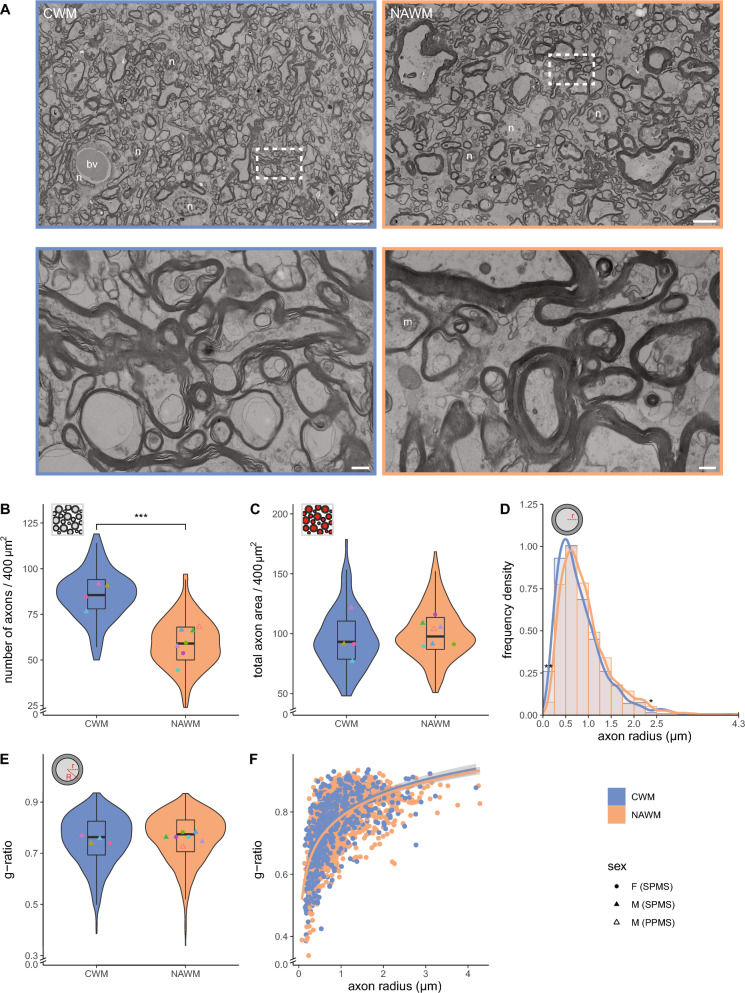
Reduced myelinated axon density in NAWM, while total myelinated axon area and g-ratio are unaffected. **A** Representative scanning transmission electron microscopic images (STEM) images of post-mortem control white matter (CGM) of control donors (from donor CM 1) normal appearing white matter (NAWM) of donors with progressive MS (from donor NAM 6). Top: overview images. Bottom: detailed images from boxed regions in top images. n = nucleus, bv = blood vessel, m = mitochondria. Scale bars represent 5 µm (top images) or 0.5 µm (bottom images). **B**, **C** Violin plots depict the number of myelinated axons (**B**) and their total area (**C**, red) in an area of 20 × 20 µm. **D** Histogram with kernel density estimation of the axon radius (r). **E** Violin plot depicts the distribution of g-ratio (r/R). Datapoints represent mean per donor (**B**, **C**, **E** ● = female (SPMS), ▲ = male (SPMS), △ = male (PPMS), color-coded, see Table 1) and boxplots show the median and inter quartile range (IQR). **F** Scatter plot showing the g-ratio versus axon radius (log-linear fit). Number of control donors (CWM) is 4, number of donors with progressive MS (NAWM) is 7, 200 myelinated axons per donor (**D**–**F**), and 25 areas of 20 × 20 µm (**B**, **C**) were analysed. Statistics were performed using an unpaired Student’s *t* test (**D**), or a linear mixed model (**B**, **C**, **E**) (*p < 0.05; **p < 0.01; ***p < 0.001)

To identify potential ultrastructural alterations in NAWM of progressive MS donors, we compared CWM from 4 non-demented control donors with NAWM from 7 donors with progressive MS (Table 1, Figs. 1A, 5A, 200 myelinated axons per donor). In NAWM there were no signs of (early) demyelination, as evidenced by similar proportions in myelin compaction scores in CWM and NAWM (Additional file 1: Fig. S1A, C). The number of myelinated axons per 400 µm^2^ was 1.5-fold lower in NAWM compared to CWM (Fig. 5A, B, NAWM: 59 ± 8.4, CWM: 86 ± 6.9, *p* < 0.001). Strikingly, myelinated axons in CWM and NAWM covered a similar cross-sectional total axon area (Fig. 5C). A rightward shift of the peak in NAWM compared to CWM, indicates enlarged myelinated axons in NAWM. More specifically, NAWM significantly contained less myelinated axons with an axon radius < 0.25 µm (Fig. 5D, *p* = 0.010) and more myelinated axons with an axon radius between 2.25 and 2.50 µm (Fig. 5D, *p* = 0.039) and tended to have more myelinated axons with an axon radius between 1.25 and 1.50 µm (Fig. 5D, *p* = 0.056). Intriguingly, in spite of the enlarged axons, no differences in the g-ratio (Fig. 5E) and scatter plots of g-ratio versus axon radius (Fig. 5F) between CWM and NAWM were noticed. Thus, NAWM contains fewer but larger myelinated axons covering a similar cross-sectional myelinated axon area as in CWM, while their g-ratio is maintained.

### Axonal mitochondrial radius does not correlate with g-ratio in NAWM

**Fig. 6:**
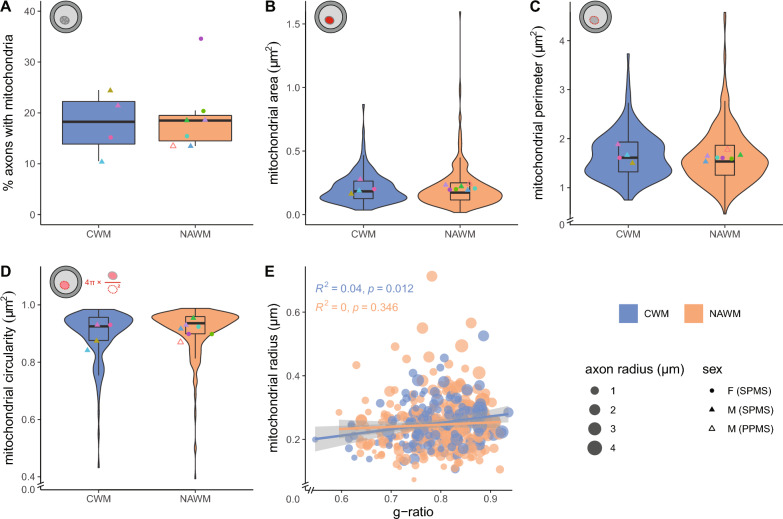
Axonal mitochondrial size does not correlate with g-ratio in NAWM. **A** Mean percentage myelinated axons with mitochondria in post-mortem control white matter (CWM) of control donors and normal appearing white matter (NAWM) tissue of progressive MS donors. **B**–**D** Analysis of cross-sectional axonal mitochondrial area (**B**), perimeter (**C**), and circularity (**D**). Icons indicate measured mitochondrial characteristics (red), violin plots depict the data distribution of all measurements, datapoints represent the mean per donor (● = female (SPMS), ▲ = male (SPMS), △ = male (PPMS), color-coded, see Table 1) and boxplots show the median and inter quartile range (IQR). **E** Scatter plot showing the correlation between mitochondria radius and g-ratio. The size of the datapoints indicates the axon radius in µm. Number of control donors (CWM) is 4, number of donors with progressive MS (NAWM) is 7, and 21–69 mitochondria per donor (dependent on the number of mitochondria in the 200 myelinated axons that were measured in Fig. 5) were analysed. Statistics were performed using an unpaired Student’s *t* test (**A**), linear mixed model (**B**–**D**), or a Pearson's r test (**E**)

To reveal whether the observed pathology of myelinated axons in NAWM affect mitochondria, we next determined mitochondria number and morphology. We counted the number of myelinated axons with at least one mitochondrion and determined the mitochondrial area, perimeter and circularity in the white matter from the same myelinated axons that were analysed in Fig. 5. From the 200 cross-sectional myelinated axons in NAWM that were analysed per donor, 11–35% contained mitochondria, which was not significantly different from CWM (Fig. 6A). The cross-sectional area, perimeter and circularity of mitochondria present in cross-sectional myelinated axons in CWM and NAWM were also similar (Fig. 6B–D). Interestingly, we observed a correlation between the mitochondrial radius and the g-ratio in CWM, while this correlation was absent in NAWM (Fig. 6E). This indicates that axons in NAWM fail to adapt their mitochondrial radius to correct for a suboptimal g-ratio.

## Discussion

**Fig. 7:**
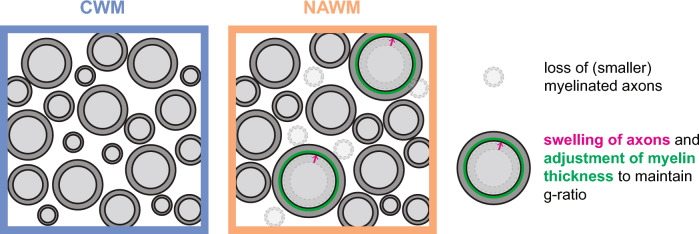
Hypothetical model explaining myelinated axon pathology in normal appearing white matter (NAWM) based on the observed ultrastructural abnormalities in myelinated axons. We argue that a loss of small calibre myelinated axons in NAWM of persons with progressive MS results in a lower myelinated axon density. To compensate for the loss of myelinated axons, remaining axons swell (pink arrow) to increase their size to cover a similar axon area. The swelled axons adjust their myelin thickness (green), explaining the unaltered g-ratio, to preserve axonal shape and limit further swelling

Conventional techniques, like MRI and immunohistochemistry, revealed signs of both early and late axonal pathology in non-demyelinated tissue of donors with progressive MS. Here, using large scale electron microscopical analysis (nanotomy) of post-mortem human brain tissue, we show that less myelinated axons in non-lesioned white matter of donors with progressive MS cover the same myelinated axon area as white matter of control donors. To compensate for the reduced density, myelinated axons in NAWM are enlarged, while retaining a similar g-ratio as myelinated axons in CWM (Fig. 7). In addition, the lack of correlation between axonal mitochondrial radius and g-ratio in NAWM indicate a disturbance in homeostasis [43] and the inability of mitochondria to adjust to a suboptimal g-ratio. In contrast to NAWM, the ultrastructural characteristics of myelinated axons and their mitochondria in the NAGM were similar compared to CGM. Hence, these ultrastructural changes in myelinated axons point to pathological alterations in (progressive) MS that may make NAWM myelin more susceptible to injury [29]. Our generated datasets are transferred into an open access database, and the systemic inclusion of higher numbers of donors that can be studied, like has been done before with nPOD [37], will not only allow to increase group size, but also makes the data directly available to others and reuse for future additional analysis, which may focus on other ultrastructural features.

Currently, information on spatial variation, including in grey versus white matter, of the g-ratio of individual axons in human brain tissue is limited. We provide systematic information regarding the g-ratio of individual myelinated axons and their mitochondria in non-lesioned grey and white matter in control and progressive MS donors. In the CNS, a g-ratio of approx. 0.77 is considered to be theoretically optimal [45]. Our analysis of individual myelinated axons in relatively large post-mortem brain tissue samples provided a g-ratio of 0.75 in CWM and 0.78 in CGM. Additionally, myelinated axons in CWM were larger than myelinated axons in CGM. MRI-based studies in control subjects report a different g-ratio. An aggregated g-ratio of 0.7 in CWM was reported using magnetization transfer with neurite orientation dispersion and density imaging (NODDI) [46] and magnetization transfer with single-shell diffusion MRI [47]. While a g-ratio of 0.71–0.85 was reported in CWM using multi-echo gradient echo myelin water imaging and NODDI [48]. Our reported g-ratio in NAWM (0.76) is higher than previously reported aggregated g-ratio’s in NAWM, i.e., 0.67 using high-gradient diffusion MRI and macromolecular tissue volume imaging [49] and 0.57 using magnetization transfer saturation and NODDI [50]. Notably, the aggregated g-ratio value obtained with MRI-based techniques depends on the used method and calculation model [51] and are more sensitive to changes or differences in large diameter axons [52]. Our analysis further revealed that the g-ratio of individually analysed myelinated axons in non-lesioned grey and white matter was similar between control donors and donors with progressive MS. This is in contrast with a recent study in post-mortem optic nerve tissue in which the g-ratio of myelinated axons in CWM was higher (0.53) than in NAWM (0.50) [18]. In addition, myelin in optic nerve NAWM was less compact and the number of mitochondria in myelinated axons was higher, while axon density and diameter were similar between optic nerve NAWM and CWM [18]. Most importantly, the authors suggest that the ultrastructural abnormalities correlated with inflammation in adjacent tissue. In contrast, we find that myelinated axons in NAWM and CWM had similar proportions of the myelin compaction score, and that the analysed areas had a similar number of non-endothelial nuclei that were evenly distributed (CWM 57 ± 15 and NAWM 42 ± 18 per median dataset area of 70 k µm^2^, *p* = 0.176). The lack of obvious signs of increased cell density by e.g., glial cell proliferation or infiltration, and decompaction, does however not exclude that the glial cells present in our dataset exhibit inflammatory alterations and/or may take up more space between myelinated axons.

The observed lower cross-sectional myelinated axon density in NAWM is consistent with (indirect) previous findings [5, 53, 54] and is likely a consequence of loss of small calibre myelinated axons, although regional differences in axon size in white matter tracts cannot be excluded. Based on the shift in frequency distribution towards larger calibre axons and a similar total axon area compared to CWM, we argue that the loss of small calibre axons is compensated by swelling of remaining myelinated axons. This is consistent with the previously reported focal swellings of axons without signs of demyelination in NAWM [19, 55]. Pathological features of swelled axons are accumulation of intra-axonal organelles and/or an increase in neurofilament spacing [55–58], most prominent in small calibre axons. The frequency of these features is most reliably analysed in 3D and/or longitudinal axonal scans. Equally reasonable, these features may resolve in NAWM as swelled myelinated axons may recover by adjusting their myelin thickness, explaining the unaltered g-ratio. This may overcome disrupted axonal conduction and preserve axonal shape by limiting further swelling as recently proposed [56]. This implies that the thickness of pre-existing myelin is not static and that mature oligodendrocytes are able to re-instate myelin membrane expansion. In control CNS, myelin sheaths continuously undergo dynamic remodelling, which is guided by neuronal activity known as OPC-based adaptive myelination [57]. Accordingly, it is tempting to suggest that the adjustment of myelin thickness in NAWM is a response of mature oligodendrocytes to altered activity of swelled axons to re-establish myelin biogenesis, i.e., oligodendrocyte-based adaptive myelination. In adult mice, newly made myelin components for e.g., myelin turnover and maintenance, are integrated at the paranodal and juxtaparanodal regions at the inner tongue [58]. Therefore, adaptive myelination by mature oligodendrocytes initiated by axonal swelling may contribute the elongation of (juxta)paranodal lengths observed in NAWM [16–19]. Furthermore, as the adjustment of myelin sheath thickness requires biosynthesis of myelin constituents, we argue that this re-instated myelination by mature oligodendrocytes causes changes in the myelin composition compared to myelin that is formed during development. This hypothesis is strengthened by reported subtle biochemical differences between NAWM and CWM myelin, and the notion that NAWM myelin is developmentally immature [20–23]. Further research is necessary to determine whether axonal swelling and subsequent adjustment of the myelin thickness indeed induces compositional changes in MS NAWM myelin.

An important remaining question is why axon density is decreased in NAWM. In murine experimental autoimmune encephalomyelitis (EAE), an experimental model mimicking inflammatory aspects of MS, axon density is also decreased in non-demyelinated white matter areas of the spinal cord, along with ongoing demyelination, axolysis and mitochondrial swelling [59]. Of note, in this EAE study, the analysed non-demyelinated areas were perilesional and may therefore harbor more extensive changes compared to white matter further away from the demyelinated areas. In acute spinal EAE lesions in mice, axonal swellings are an early ultrastructural sign of damage that precede demyelination [55]. Interestingly, some of these swollen axons recovered spontaneously, indicating that axonal swelling can be reversible. Therefore, it is tempting to suggest that by adjusting myelin thickness the myelinated axons may recover from axonal swelling. The potential consequence of axonal swellings is a decrease in conduction velocity [60]. Axonal swelling is thought to be triggered by mitochondrial pathology induced by macrophage-derived reactive oxygen and nitrogen species [55]. Although we did not observe ultrastructural differences between mitochondria in NAWM and CWM in our cross-sectional analysis, we did find that the correlation between the cross-sectional mitochondrial radius and g-ratio shown at homeostatic conditions [43], was lost in NAWM of donors with progressive MS. This may point at the inability of mitochondria to adapt to the increased myelin thickness of enlarged axons [43]. Due to the limitations of EM imaging, we can however not exclude axonal mitochondrial swelling in NAWM and/or alterations in number, localisation and proper functioning of axonal mitochondria.

Apart from analysis of end stage post-mortem brain tissue, limitations of our study are that the control donors were older than the MS donors and mainly males. While sex differences in g-ratio occur during adolescence [61], the data on the influence of sex on g-ratio in adults are conflicting. One MRI-based study showed that the whole-brain g-ratio is not influenced by sex [46], while other studies observed a smaller g-ratio, and thus higher myelin content, in whole-brain of females [62]. In our data, no clear segregation based on sex within the group of MS donors for most tested parameters was detected. In contrast, axon density decreases and g-ratio increases with age [46, 62, 63]. Thus, estimation of myelin volume fraction and axonal volume fraction of MRI images indicate that aggregate g-ratio values increase from middle age in most regions examined. In mouse optic nerve, using three-dimensional EM, ageing axons are larger and have thicker myelin and larger mitochondria [64]. Hence, the age difference between our control and MS donors might underrepresent the lower axon density and larger calibre axons observed in NAWM compared to CWM.

In summary, our findings indicate a loss of small calibre myelinated axons in NAWM and we argue that this is loss is compensated by swelling of remaining axons that adjust their myelin thickness to maintain their g-ratio without adaptation of mitochondria size (Fig. 7). These subtle ultrastructural changes in NAWM point to pathological alterations in progressive MS. As in EAE, axonal swelling might be spontaneously reversible and/or an ultrastructural sign of damage that precedes demyelination. More insight in potential (biochemical) abnormalities of myelin on (recovered) swelled axons may uncover subtle disruption in myelin structure that for example contributes to the increased risk of MS myelin to (ongoing) degeneration and/or elicit (secondary) pathological inflammation. Furthermore, identification of (axonal) signals that re-instate either myelin biogenesis or the response of mitochondria to the adapted g-ratio may lead to novel and more effective therapeutic strategies to halt white matter lesion formation in MS.

## Supplementary Information

**Additional file 1: Fig. S1**. CM and NAM have similar proportions of myelin compaction scores. **A** Schematic and representative scanning transmission electron microscopic image of cross-sectional myelinated axons with myelin compaction scores ranging from 1–5. The percentage indicates the estimated compact myelin area. Scale bars represent 0.5 µm. **B** Average proportions of myelin compaction scores in control grey matter (CGM) of control donors and normal appearing grey matter (NAGM) of donors with progressive MS. **C** Average proportions of myelin compaction scores in control white matter (CWM) of control donors and normal appearing white matter (NAWM) of donors with progressive MS. Statistics were performed using a general linear multivariate model test (SPSS 28, not significant).

**Additional file 2: Fig. S2**. Dendritic mitochondria in CGM and NAGM are similar. **A** Representative scanning transmission electron microscopic cross-sectional image of a dendrite with a mitochondrion in post-mortem grey matter brain tissue. Scale bar represents 0.25 µm. **B** Mean percentage of dendrites with mitochondria in post-mortem control grey matter (CGM) of control donors and normal appearing grey matter (NAGM) tissue of donors with progressive MS. **C**–**E** Analysis of cross-sectional dendritic mitochondrial area, perimeter, and circularity. Icons indicate measured mitochondrial characteristics, violin plots depict the data distribution of all measurements, data points represent mean per donor, ▲ = male, △ = male, color-coded, see Table 1), and boxplots show the median and inter quartile range. Number of control donors is 4 and number of donors with progressive MS is 6. 200 dendrites per donor were analysed of which the number of dendritic mitochondria ranged from 44–101. Statistics were performed using an unpaired Student’s *t* test or linear mixed model (not significant).

## Data Availability

The datasets generated and analysed in the current study are available at full resolution at nanotomy.org.

## Abbreviations

CGM: Control grey matter
CM: Control matter
CNS: Central nervous system
CWM: Control white matter
EAE: Experimental autoimmune encephalomyelitis
EM: Electron microscopy
MRI: Magnetic resonance imaging
MS: Multiple sclerosis
MTR: Magnetisation transfer imaging
NAGM: Normal appearing grey matter
NAM: Normal appearing matter
NAWM: Normal appearing white matter
NODDI: Neurite orientation dispersion and density imaging
nPOD: Network for pancreatic organ donors with diabetes
OPC: Oligodendrocyte progenitor cell
PPMS: Primary progressive multiple sclerosis
ROS: Reactive oxygen species
RRMS: Relapsing remitting multiple sclerosis
SPMS: Secondary progressive multiple sclerosis
STEM: Scanning transmission electron microscopy
