# Supplementary figures and images for: Pathological ultrastructural alterations of myelinated axons in normal appearing white matter in progressive multiple sclerosis
Source: Acta Neuropathol Commun. 2023 Jun 20;11:100. doi: 10.1186/s40478-023-01598-7 (PMC10283269; doi:10.1186/s40478-023-01598-7)

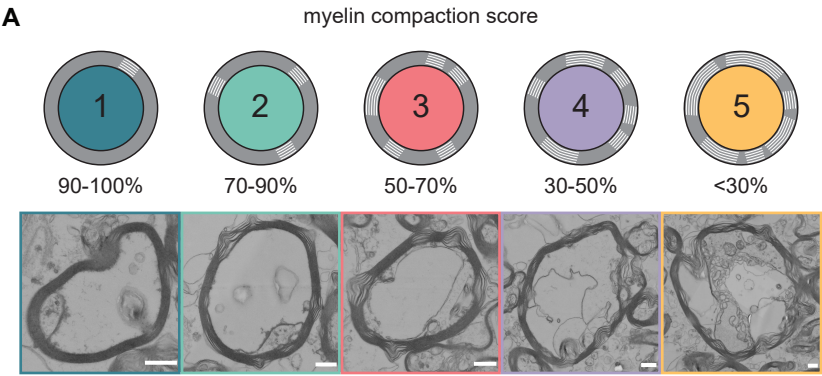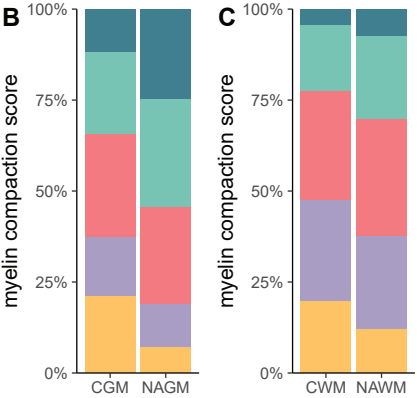

Supplement: Supplementary file 1 — Additional file 1: Fig. S1. CM and NAM have similar proportions of myelin compaction scores. A Schematic and representative scanning transmission electron microscopic image of cross-sectional myelinated axons with myelin compaction scores ranging from 1–5. The percentage indicates the estimated compact myelin area. Scale bars represent 0.5 µm. B Average proportions of myelin compaction scores in control grey matter (CGM) of control donors and normal appearing grey matter (NAGM) of donors with progressive MS. C Average proportions of myelin compaction scores in control white matter (CWM) of control donors and normal appearing white matter (NAWM) of donors with progressive MS. Statistics were performed using a general linear multivariate model test (SPSS 28, not significant). [file 40478_2023_1598_MOESM1_ESM.pdf]

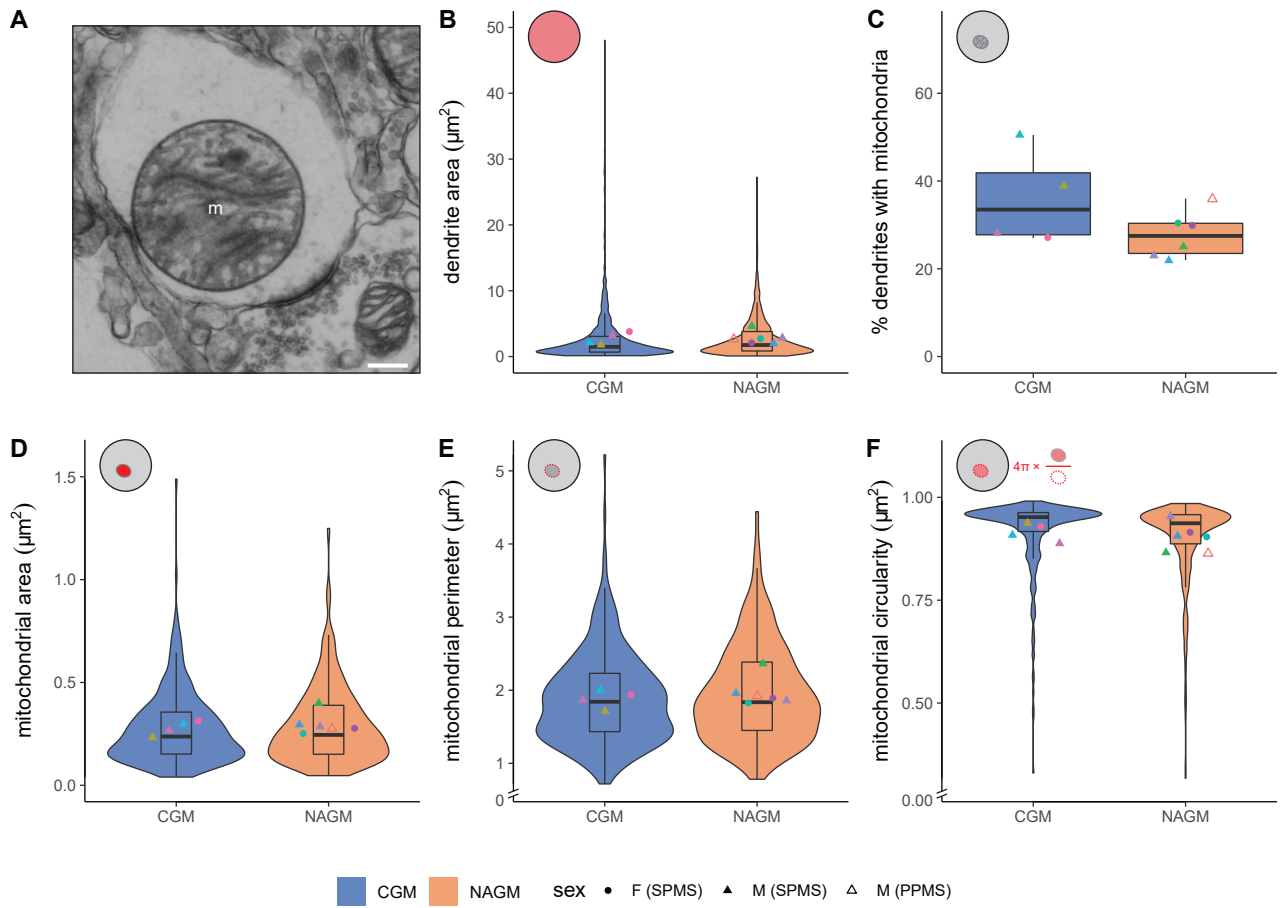

Supplement: Supplementary file 2 — Additional file 2: Fig. S2. Dendritic mitochondria in CGM and NAGM are similar. A Representative scanning transmission electron microscopic cross-sectional image of a dendrite with a mitochondrion in post-mortem grey matter brain tissue. Scale bar represents 0.25 µm. B Mean percentage of dendrites with mitochondria in post-mortem control grey matter (CGM) of control donors and normal appearing grey matter (NAGM) tissue of donors with progressive MS. C–E Analysis of cross-sectional dendritic mitochondrial area, perimeter, and circularity. Icons indicate measured mitochondrial characteristics, violin plots depict the data distribution of all measurements, data points represent mean per donor, ▲ = male, △ = male, color-coded, see Table 1), and boxplots show the median and inter quartile range. Number of control donors is 4 and number of donors with progressive MS is 6. 200 dendrites per donor were analysed of which the number of dendritic mitochondria ranged from 44–101. Statistics were performed using an unpaired Student’s t test or linear mixed model (not significant). [file 40478_2023_1598_MOESM2_ESM.pdf]
